# Eye movement desensitization and reprocessing (EMDR) therapy in the treatment of depression: a matched pairs study in an inpatient setting

**DOI:** 10.1002/brb3.342

**Published:** 2015-04-30

**Authors:** Michael Hase, Ute Mirian Balmaceda, Adrian Hase, Maria Lehnung, Visal Tumani, Christian Huchzermeier, Arne Hofmann

**Affiliations:** 1 Diana KlinikDahlenburger Str. 2a, D-29549, Bad Bevensen, Germany; 2 Department of Organisational Psychology, University of GroningenGroningen, The Netherlands; 3 Clinical PsychologistKieler Str. 74-76, D-24340, Eckernfoerde, Germany; 4 Department of Psychiatry, Ulm UniversityLeimgrubenweg 12-14, D-89075, Ulm, Germany; 5 ZiPNiemannsweg 147, D-24105, Kiel, Germany; 6 EMDR-InstituteDolmannstr. 86 b., D-51427, Bergisch Gladbach, Germany; 7 Lueneburg Center of StressmedicineIm Kamp 9, D-21335, Lueneburg, Germany

**Keywords:** AIP model, depression, EMDR, pathogenic memory, stressful life experiences

## Abstract

**Background:**

Depression is a severe mental disorder that challenges mental health systems worldwide as the success rates of all established treatments are limited. Eye Movement Desensitization and Reprocessing (EMDR) therapy is a scientifically acknowledged psychotherapeutic treatment for PTSD. Given the recent research indicating that trauma and other adverse life experiences can be the basis of depression, the aim of this study was to determine the effectiveness of EMDR therapy with this disorder.

**Method:**

In this study, we recruited a group of 16 patients with depressive episodes in an inpatient setting. These 16 patients were treated with EMDR therapy by reprocessing of memories related to stressful life events in addition to treatment as usual (TAU). They were compared to a group of 16 controls matched regarding diagnosis, degree of depression, sex, age and time of admission to hospital, which were receiving TAU only.

**Results:**

Sixty-eight percent of the patients in the EMDR group showed full remission at end of treatment. The EMDR group showed a greater reduction in depressive symptoms as measured by the SCL-90-R depression subscale. This difference was significant even when adjusted for duration of treatment. In a follow-up period of more than 1 year the EMDR group reported less problems related to depression and less relapses than the control group.

**Conclusions:**

EMDR therapy shows promise as an effective treatment for depressive disorders. Larger controlled studies are necessary to replicate our findings.

## Introduction

Given its frequency and severity, depression is a severe challenge to mental health systems worldwide, and this challenge is increasing. The World Health Organization (2012) has named depression as one of the most frequent and disabling diagnoses in the world, affecting at least 350 million people worldwide, almost one million of whom commit suicide each year (Murray and Lopez 1996; Greden 2001). Psychotherapeutic interventions have a long tradition in the treatment of depression. Several reports show that psychotherapeutic interventions can be helpful, not only in light and moderate depression but also in cases of severe chronic depression (Nemeroff et al. 2003). However, relapse rates are still high, even in patients who respond to the different forms of psychotherapeutic treatment. In fact, 1 year after discontinuation of psychotherapy for acute depression, the relapse rate was 29%, and increased to 54% after 2 years (Vittengl et al. 2007).

Chronic and acute stressors are well-established contributors to depression and can even trigger the onset of depressive episodes (Heim and Nemeroff 2001; McFarlane 2010). First episodes of depression are often more closely related to a specific psychosocial stressor than later episodes, while later episodes of depression can be triggered by far smaller events, or even occur without any noticeable stressor (Post 1992). The strong influence of adverse life events on the development of depression is also apparent in a meta-analysis conducted by Risch et al. (2009), where the only risk factor significantly correlated with depression was the occurrence of stressful life events. The presence of a serotonin transporter gene polymorphism alone, or even in combination with adverse life events, was not significantly correlated with the occurrence of depressive episodes. These results are in agreement with the studies showing that adverse and traumatic life events seem to have a close relationship with the occurrence of depressive episodes in dose–response and temporal terms (Wise et al. 2001). As indicated by Heim et al. (2004) there are neurobiologically different subtypes of depression depending on the presence or absence of early adverse experience, likely influencing treatment response in depression. It seems that depressive disorders may have more in common with PTSD than is reflected in the current psychotherapeutic treatment approaches for depression.

Eye Movement Desensitization and Reprocessing therapy (EMDR) is widely recognized as an empirically supported treatment for PTSD (Bisson and Andrew 2007). According to the new WHO practice guidelines (World Health Organization 2013) trauma-focused CBT and EMDR are the only psychotherapies recommended for children, adolescents, and adults with PTSD. In addition, a recent meta-analysis evaluating 26 randomized controlled trials (Lee and Cuijpers 2013) has demonstrated the significant effects of the eye movement component in the reduction in emotional distress.

EMDR therapy is guided by the adaptive information processing (AIP) model (Shapiro 2001). One of the key tenets of the AIP model is that dysfunctionally stored and not fully processed memories are the cause of a number of mental disorders, including PTSD, adjustment disorders, some forms of depression, and anxiety disorders (Shapiro 2014). Adverse life experiences are posited to have effects comparable to major trauma. In support of this thesis are data from a survey of 832 people (Mol et al. 2005) indicating that life events can generate at least as many PTSD symptoms as traumatic events. For events from the past 30 years the PTSD scores were higher after life events than after traumatic event.

Systematic studies have demonstrated the effects of EMDR therapy on PTSD-related depression. In a randomized clinical trial, van der Kolk and colleagues compared the effectiveness of fluoxetine treatment with EMDR therapy and placebo in a PTSD population (van der Kolk et al. 2007). After the intervention the EMDR group had significantly lower BDI scores than the fluoxetine group. This led the authors of this study to conclude that, “once the trauma is resolved, other domains of psychological functioning appear to improve spontaneously”.

This finding was echoed by other controlled studies such as a study by Power and colleagues, where PTSD patients were treated with either CBT or EMDR therapy (the control group consisted of patients on a waiting list). Both intervention groups experienced significant improvements in both PTSD and depression symptoms that where maintained at follow-up 6 months later although EMDR therapy was more efficient in reducing depression symptoms (Power et al. 2002). The amelioration of depressive symptoms following EMDR treatment of memories that patients experience as traumatic seems not to be limited to PTSD patients alone. In a controlled study Wilson et al. (1995) treated a group suffering from traumatic memories. Although only 54% of these patients were diagnosed with PTSD, all of them benefited from EMDR treatment, as evidenced by significant improvements in their depressive symptoms. These benefits were maintained at 15-month follow-up (Wilson et al. 1997). The observation that depressive symptoms seem to be strongly linked with noncriterion A events is also supported by a number of case reports where depressive patients were successfully treated with EMDR therapy either alone or as an adjunct to other therapy approaches (Manfield 1998; Tinker and Wilson 1999; Sun et al. 2004; Broad and Wheeler 2006; Shapiro 2009; Rosas Uribe et al. 2010; Grey 2011). For instance, two adolescents with major depression alone were successfully treated with EMDR therapy in three and seven sessions respectively and treatment results were stable at 3 months follow-up (Bae et al. 2008). In both cases, EMDR was used successfully focusing on events related to change in or loss of relationships. Such events also seem to be a specific risk factor for depressive disorders. In a large, retrospective study, losses, separation events, and humiliating events were strongly linked to an increased risk of depressive episodes (Kendler et al. 2003). Twelve sessions of EMDR therapy administered during a 1 month period was also reported to result in the remission of both anxiety and depression in a patient diagnosed with major depressive disorder and panic disorder (Grey 2011). In addition, a study comparing cognitive behavioral therapy (CBT) alone and CBT plus EMDR therapy reported significantly more remission of depression in the latter condition (Hofmann et al. 2014).

As no controlled studies have been published using EMDR as a psychotherapeutic approach for patients diagnosed with depression without comorbid PTSD, the present pilot study was conducted as an additional step on a road to more rigorous research. On the basis of the above-mentioned literature it was hypothesized that EMDR would be more effective than treatment as usual (TAU) in treating depressive disorders. The depression subscale of the Symptom Checklist 90 revised (SCL-90-R) was selected as an objective measure of the current severity of depression and is described in more detail below. In particular, we predicted that TAU + EMDR treatment would be more effective than TAU alone in reducing scores on the SCL-90-R depression subscale (Hypothesis 1) and the SCL-90-R global severity index (GSI) (Hypothesis 2).

## Materials and Methods

To gain more systematic insight into the effects of EMDR treatment on depressive patients we recruited 16 inpatients at a psychodynamically based clinic who agreed to participate in the study. These patients received EMDR therapy in relation to memories of nontraumatic stressful life events and treatment as usual (TAU). In parallel we identified 16 patients matched for time of admission, gender, age, and diagnosis.

### Eye movement desensitization and reprocessing therapy (EMDR)

EMDR therapy is a manualized 8-phase psychotherapy approach that was developed by Shapiro (2001) based on the Adaptive Information Processing (AIP) model. The eight phases of EMDR therapy consist of client history and treatment planning (Phase 1), preparation (Phase 2), assessment (Phase 3), desensitization and reprocessing (Phase 4), installation (Phase 5), body scan (Phase 6), closure (Phase 7), and reevaluation (Phase 8). A key component of EMDR therapy is bilateral stimulation, usually therapist-guided eye movements, which initiate information processing on the targeted memory. This component has been found to significantly contribute to positive treatment effects (Lee and Cuijpers 2013).

### Participants

The sample consisted of 32 inpatients at a rehabilitation clinic for psychosomatic medicine and psychotherapy. They were diagnosed as suffering from a mild-to-moderate depressive episode or a mild-to-moderate depressive episode related to recurrent depression according to ICD-10 criteria. Sixteen patients gave their informed consent to receive EMDR therapy in addition to treatment as usual (study group). A further 16 patients were matched regarding time of admission, gender, age, and diagnosis and received treatment as usual. These patients acted as a control group. Both groups were comparable regarding the severity of depression as measured by the SCL-90-R depression scale.

The mean age of the entire sample was 46.41 years (SD = 9.06), while the mean age of the control group was 49.5 years (SD = 7.47) and the mean age of the study group was 43.31 years (SD = 9.67). The age differences between the two treatment conditions were not statistically significant (*P *=* *0.05). Six of the patients who agreed to receive EMDR were female, 10 were male. The gender distribution in the control group was matched. Nine of the patients in the EMDR group were suffering from a recurrence of major depression (F 33.2) and seven were suffering from a depressive episode (F 32.2) at the time of admission. As the control group was matched regarding diagnosis the distribution of single depressive episode and recurrent depression was the same as in the study group. The study group stayed for 45.81 days in treatment (SD = 8.91), while control group patients stayed for 39.37 days in treatment (SD = 5.64). Nine patients in the study group and 10 patients in the control group were on antidepressant medication at the time of admission. This difference was not statistically significant (Fisher's exact test, *P *=* *0.716). Four patients in the control group and one patient in the study group were taking more than one drug. One patient in the control group was on carbamazepine for his epilepsy. The types of antidepressant medication and their distribution are given in Table 1.

**Table 1:** Types of antidepressant medication

	SSRI	NaSSA	Other
Study group	2	4	3
Control group	5	2	3

SSRI, Selective Serotonine Re-Uptake Inhibitors; NaSSA, Noradrenergic and Specific Serotonergic Antidepressants.

### Study procedure

The study group patients were all assessed and treated by an experienced EMDR therapist whose fidelity had been previously assessed. They were informed about the research-based use of EMDR therapy in the treatment of depression and gave informed consent after receiving extensive information. As this was more a proof of concept study in preparation of a larger randomized controlled study it seemed favorable to match a control group in order to gather preliminary data. We decided to administer EMDR therapy by the most experienced EMDR therapist. So the patients routinely allocated to this therapist were included in the study group if they met the criteria and decided to participate. The control group was formed by selecting patients matching the study group participants regarding diagnosis, severity of depression, sex, age, and time of admission to hospital. The diagnostic procedures with the control group were limited to standard clinic procedures. Diagnostic procedures at admission and before discharge thus consisted of the Symptom Checklist 90 Items revised version (SCL-90-R) (Derogatis et al. 1973) with all patients and the Beck depression Inventory (BDI) (Hautzinger et al. 2006) in addition in the study group. With both groups the Global Severity Index (GSI) and the depression scale of the SCL-90-R were analyzed. Because of limited resources the BDI was only used with the study group patients. Testing was conducted by an independent assessor using the pc-based Hogrefe Test System. The assessor was not aware of the treatment condition.

EMDR therapy sessions were administered once a week if a memory could be reprocessed completely in a session. In the case of incomplete reprocessing a second EMDR therapy session was scheduled in the same week.

The patients in both control group and study group were reassessed 12 to 16 months after end of in-patient treatment. They were asked to fill out a self-report form asking for the number of depressive episodes since termination of treatment, ongoing treatment (medication, psychotherapy or in-patient treatment), and periods of sick leave from work.

### Treatment setting

Treatment as usual consisted of psychodynamic psychotherapy in one-to-one sessions, group therapy sessions and a course of five group sessions of psychoeducation and improvement of coping with depression. Patients in both groups also received sports therapy and relaxation therapy. The patients in the study group received EMDR therapy sessions of 60 minutes duration on disturbing memories related to the onset and course of their depressive disorder mostly on a weekly basis. These were mostly memories of adverse life events below PTSD criterion A threshold. All phases of EMDR therapy were conducted according to standardized procedures (Shapiro 2001) which involved processing past memories, current triggers and future needs. Adverse effects were monitored by observation during reprocessing and by active questioning at the end of an EMDR-session and at the beginning of the next session.

The average numbers of therapy sessions in the control group were 6.5 (SD = 2.5) individual and 7 (SD = 3.9) group psychotherapy sessions. In the study group the average numbers of therapy sessions were 5.6 (SD = 2.4) individual and 7.6 (SD = 4.5) group psychotherapy sessions. The number of EMDR therapy sessions was 4.6 (SD = 2.4). Descriptive statistics for age, total treatment duration in days, number of individual therapy sessions received, number of group therapy sessions received, and number of EMDR therapy sessions received are given in Table 2.

**Table 2:** Descriptive comparisons between group means, standard deviations in parentheses

	Age (years)	Days spent in treatment	Number of individual therapy sessions	Number of group therapy sessions	Number of EMDR sessions
Study	43.31 (9.67)	45.81 (8.91)	5.60 (2.40)	7.60 (4.50)	4.60 (2.40)
Control	49.50 (7.47)	39.37 (5.64)	6.50 (2.50)	7.00 (3.90)	0.00 (0.00)

### Statistical analysis

The data were analyzed with IBM SPSS Statistics 20, IBM Corp., Armonk, New York. A two-group multivariate analysis of covariance (MANCOVA) was used to analyze group differences between the control group and the study group on GSI and SCL-90 R depression subscale change scores, controlling for the number of therapy sessions received. A simple contrast with the control group as reference group was used to more closely examine possible differences between the groups. In addition bivariate correlations were computed for the study group between the amount of EMDR therapy received and the change in GSI, SCL-90 R Depression subscale and BDI scores.

## Results

Results consisted of posttreatment changes regarding psychological testing and behavioral changes, for example, use of medication as well as follow-up data, for example, relapse or absence from work.

### Psychopathological changes

In the study group 11 of 16 patients showed full remission at the end of treatment indicated by a BDI score of 12 or less. This means that 68% of the patients treated with EMDR showed full remission at the end of treatment. The study group showed a greater reduction in depressive symptoms as measured by the SCL-90-R depression subscale. This difference was significant even when adjusted for duration of treatment.

Descriptive statistics for the baseline and posttreatment scores of both groups are given in Table 3. At baseline, the groups did not differ significantly in their GSI (*t*(30) = 0.964, *P *=* *0.343) or SCL-90 R Depression (*t*(30) = 0.896, *P *=* *0.378) scores.

**Table 3:** Descriptive data for baseline and posttreatment scores on Global Severity Index (GSI), SCL-90R depression subscale (Depr), and Beck Depression Inventory (BDI)

	Mean control group (SD), *n* = 16	Mean study group (SD), *n* = 16
GSI (baseline)	1.28 (0.59)	1.12 (0.33)
GSI (posttreatment)	1.16 (0.91)	0.42 (0.32)
Depr (baseline)	23.69 (9.34)	20.94 (7.97)
Depr (posttreatment)	19.61 (13.18)	6.94 (6.72)
BDI (baseline)		21.13 (7.67)
BDI (posttreatment)		7.81 (6.21)

SD, standard deviation.

**Figure 1:**
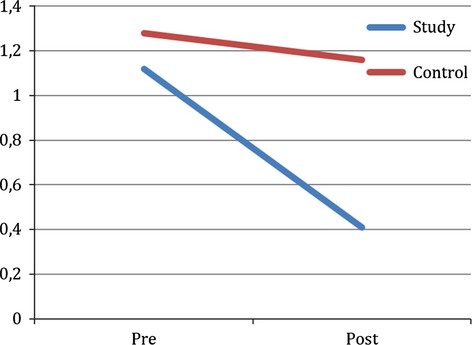
SCL-90-R GSI score change pre- versus posttreatment in study (EMDR therapy) and control (treatment as usual) group indicating a significant difference regarding outcome measure between groups (*P *=* *0.015).

**Figure 2:**
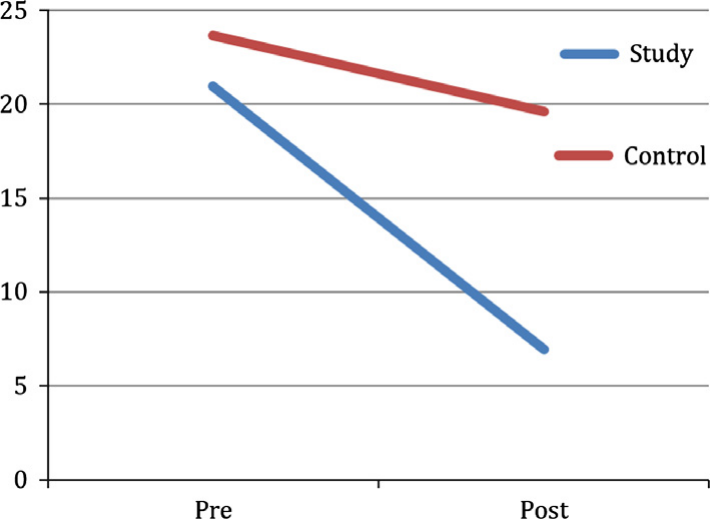
SCL-90-R Depression subscale score change pre- versus posttreatment in study (EMDR therapy) and control (treatment as usual) group indicating a significant difference regarding outcome measure between groups (*P *=* *0.04).

The MANCOVA yielded no significant effect of group (*F*(2, 28) = 3.335, *P *=* *0.05, *η*² = 0.192) at the 95% confidence level. A simple contrast showed that the decrease in GSI score (Fig.1) was significantly larger in the study group than in the control group (contrast estimate = −0.490, *P *=* *0.015, *d *=* *1.18). The same was the case for the change in SCL-90 R Depression subscale score (contrast estimate = −7.709, *P *=* *0.047, *d *=* *1.02) as can be seen in Fig.2. The observed power was reported to be 58.3%.

Pearson correlations were computed between the total number of therapy sessions received and the changes in GSI and SCL-90 R depression subscale scores. Number of therapy sessions was significantly correlated with change in SCL-90 R depression subscale score (*r *=* *0.401, *P *=* *0.023) and with change in GSI score (*r *=* *0.379, *P *=* *0.033), thus justifying its use as a covariate. Furthermore, the amount of EMDR sessions that the subjects received was correlated with the change in GSI, SCL-90 R depression subscale and BDI scores for the subjects in the EMDR group.

At termination of treatment 12 patients in the control group and seven patients in the study group were still on antidepressant medication. One patient in the study group was put on antidepressant medication during the clinic stay. Three patients in the study group terminated medication because of significant improvement, while this was only the case with one patient in the study group. The number of patients on medication at the beginning and end of treatment can be found in Table 4.

**Table 4:** Antidepressant medication at beginning and end of treatment (number of patients)

	Antidepressant medication pretreatment	Antidepressant medication posttreatment	Medication discontinued
Study group (*n* = 11)	9	7	3
Control group (*n* = 9)	10	11	1

### Adverse effects and safety

Adverse effects were not reported during reprocessing or during reevaluation. This showed that the EMDR therapy sessions were well tolerated by the patients. Hyperarousal was hardly observed within the sessions. Intensive affect was observed in some sessions but could be managed and reprocessed. The timeframe of 60 min per session was sufficient for processing of most of the targeted memories.

### Follow-up data

A total of 20 patients sent back the self-report form at follow-up. In the study group, 11 of 16 patients responded. Two patients had moved in the meantime and three patients failed to report back without any explanation. In the control group 9 of 16 patients responded and seven patients failed to report back without any explanation.

The impact of EMDR on relapses was of course of interest. Of the 11 patients in the study group reporting back at follow-up, only three had experienced a relapse during the follow-up period. Eight patients reported the absence of depression. In the control group nine patients reported back at follow-up, six of them reporting another depressive episode during the follow-up period. Fisher's exact test showed no significant differences in the distributions of the relapses (*P *=* *0.175), probably due to small numbers. A total of 20 subjects gave feedback on absences from work due to their psychological condition approximately 1 year after returning from therapy. Table 5 shows the distribution regarding absence from work across the four categories on the questionnaire. Due to departures from normality, a nonparametric independent-samples test was used to assess the distribution differences between the two groups. The result was significant at *P *=* *0.003, indicating that the distributions of work absences were significantly different in favor of the study group.

**Table 5:** Work status in the year after therapy (number of patients)

	Not absent from work	Absent from work for <7 days	Absent from work for 7 to 30 days	Absent from work for >30 days
Study group (*n* = 11)	9	1	0	1
Control group (*n* = 9)	1	0	3	5

At follow-up seven members of the control group and four members of the study group were still on antidepressant medication as shown in Table 6.

**Table 6:** Medication at follow-up (number of patients)

	Antidepressant medication	No Antidepressant Medication
Study group (*n* = 11)	4	7
Control group (*n* = 9)	8	1

## Discussion

EMDR therapy is based on the AIP model. One of the key assumptions of the AIP model is that dysfunctionally stored (disturbing) memories are the cause of a number of mental pathologies, including PTSD and other trauma-based disorders and also some forms of depression. A number of controlled studies have also found EMDR therapy to be effective for disorders that are linked with traumatic events (Shapiro and Maxfield 2002). The AIP model postulates that if the patient's dysfunctionally stored stressful memories are reprocessed and finally integrated adaptively into the memory networks, the associated psychopathology subsides (Shapiro 2001). This suggests that adding EMDR therapy to the treatment as usual could show some benefit.

Different EMDR therapy strategies were used depending on case history and present status. One of the most useful targeting strategies was to focus on the events that had precipitated the (last or worst) depressive episode(s) in line with Shapiro's approach to focus on the events that set the psychopathology in motion (Shapiro 2001). Most of these events did not fit into the PTSD criterion A category but could be classified as meaningful disturbing memories, which are considered to be effective targets for EMDR therapy reprocessing (Frustaci et al. 2010). Interestingly these memories often focused on life events that are known to be related to the onset or continuation of depression (Kendler et al. 2003). Many of these memories were memories of losses, separations and humiliations, the very types of memories that seem to be connected to the occurrence of depressive disorders. This fits well with studies that have shown that victims of adverse life events do not remember Criterion A events as being “more traumatic” than other disturbing life events (Gold et al. 2005). Working with negative beliefs and the memories on which they were founded was another successful strategy in some of the patients.

Adverse effects were not reported during reprocessing or during reevaluation. This showed that the EMDR therapy sessions were well tolerated by the patients. Hyperarousal was hardly observed within the sessions. One could hypothesize that hyperarousal as a core symptom of PTSD is associated with problems in memory reprocessing, but is seldom observed in processing of nontraumatic pathogenic memories. This is in line with the observation of Frustaci et al. (2010).

Support was found for both hypotheses, namely that TAU + EMDR were more effective than just TAU in reducing SCL-90-R depression subscale and GSI scores. The pre- to posttreatment reduction in the SCL-90-R depression subscale scores in the study group was significantly larger than the decrease in the control group. Although the patients in the study group stayed longer at the clinic, this did not explain the difference in the drop in SCL-90-R depression scale score. These effects persisted after statistical adjustment for the longer therapy duration in the control group. Furthermore, the significant difference in distributions of days absent from work indicates that EMDR may have helped patients to function more effectively and cope better with job demands and stressors after return from therapy.

Regarding the problem of missing data at follow-up, it seems prudent not to draw too many conclusions. The existing data show a trend, albeit not significant, toward less relapse in the study group which could be related to a deeper impact of EMDR by processing pathogenic memories (Centonze et al. 2005). It would be interesting to research this hypothesis in a randomized controlled study. The missing values in both groups lend even less power to the detection of differences in relapse rates and thus it remains unclear whether the lack of significance was due to there being no underlying difference between the groups, or due to power issues. One could assume, that EMDR in addition to TAU delivers more sustainable, long-term therapy benefits than TAU alone. It could thus reduce absence from work and health care expenditure after therapy completion. Of course, these hypotheses need to be tested in randomized controlled trials with larger samples.

Naturally, there are several limitations to this study. First of all, the low sample size limits the generalizability of the results and calls for replication to see whether the present results persist in a larger sample. With only 16 subjects per group, one cannot be entirely sure that the positive effects were simply due to chance. Another limitation concerns the potential third outcome measure. Unlike the depression scale and the GSI scores, the BDI scores could not be examined in the analysis as this scale was used with study group patients only. This limits the comparison on full remission between groups as full remission was defined by a BDI score < 12 and the BDI was only conducted with study group patients for reasons explained above. Further limitations of this matched pairs study are the absence of a randomized design, the absence of the BDI in TAU and the absence of a structured interview and testing at follow-up. But the aim of this study was to find out whether EMDR therapy would be effective as an addition to treatment as usual.

## Conclusion

This study provides evidence that EMDR therapy has significant positive effects in the treatment of depressive episodes and recurrent depression. In a previous study, similar results were obtained (Hofmann et al. 2014). In that study, EMDR therapy was added to cognitive behavioral psychotherapy (CBT) compared to CBT as TAU in an outpatient setting. The addition of a mean of seven session of EMDR therapy resulted in a significant difference in symptom decline, with 18 of 21 patients achieving remission of depression in the EMDR + CBT condition compared to eight of 21 in CBT alone. Future research should therefore examine the effect of EMDR therapy alone compared to the most effective treatment available.

But the direction of research should not be limited on depressive disorders but expended to the affective spectrum. Novo et al. (2014) studied EMDR therapy on twenty DSM-IV bipolar I and II patients with subsyndromal mood symptoms and a history of traumatic events. These were randomly assigned to EMDR therapy or treatment as usual. Evaluations of affective symptoms, symptoms of trauma, and trauma impact were carried out by a blind rater at baseline, 2 weeks, 5 weeks, 8 weeks, 12 weeks, and at 24 weeks follow-up. Patients in the treatment group showed a statistically significant improvement in depressive and hypomanic symptoms, symptoms of trauma, and trauma impact compared to the treatment as usual group after intervention. This pilot study suggests that EMDR therapy may be an effective and safe intervention to treat subsyndromal mood and trauma symptoms in traumatized bipolar patients.

We hope to initiate more research on this promising approach to alleviate suffering in such a common and debilitating disorder as recurrent depression. Despite the limitations of this study, these results combined with the results of other pilot studies should spark enough interest to generate further research and improve the state of knowledge on the efficacy of EMDR treatment in depression and affective disorders in general.

## Disclosure Statement

Dr. Michael Hase is an EMDR Institute trainer providing training in EMDR. Dr. Arne Hofmann is head of the EMDR Institute Germany providing training in EMDR. Dr. Maria Lehnung, and Dr. Visal Tumani are staff of the EMDR Institute Germany. The other authors have no relevant financial incentives to declare.
